# A Comparison of Three Empirical Reliability Estimates for Computerized Adaptive Testing (CAT) Using a Medical Licensing Examination

**DOI:** 10.3389/fpsyg.2018.00681

**Published:** 2018-06-28

**Authors:** Dong Gi Seo, Sunho Jung

**Affiliations:** ^1^Department of Psychology, Hallym University, Chuncheon, South Korea; ^2^School of Management, Kyung Hee University, Seoul, South Korea

**Keywords:** reliability, item response theory (IRT), computerized adaptive testing, measurement, classical test theory

## Abstract

Arithmetic mean, Harmonic mean, and Jensen equality were applied to marginalize observed standard errors (OSEs) to estimate CAT reliability. Based on different marginalization method, three empirical CAT reliabilities were compared with true reliabilities. Results showed that three empirical CAT reliabilities were underestimated compared to true reliability in short test length (<40), whereas the magnitude of CAT reliabilities was followed by Jensen equality, Harmonic mean, and Arithmetic mean when mean of ability population distribution is zero. Specifically, Jensen equality overestimated true reliability when the number of items is over 40 and mean ability population distribution is zero. However, Jensen equality was recommended for computing reliability estimates because it was closer to true reliability even if small numbers of items was administered regardless of the mean of ability population distribution, and it can be computed easily by using a single test information value at θ = 0. Although CAT is efficient and accurate compared to a fixed-form test, a small fixed number of items is not recommended as a CAT termination criterion for 2PLM, specifically for 3PLM, to maintain high reliability estimates.

## Introduction

Nicewander and Thomasson (1999) applied *Arithmetic, Harmonic*, and *Jensen's inequality* methods to marginalize test information for estimating IRT reliability estimates in computerized adaptive testing (CAT). However, the items were drawn from item banks containing an average of 80 items per test, which were longer than practical CAT set up. In addition, many practical assessment programs often used interchangeably three IRT reliabilities (*Arithmetic, Harmonic*, and *Jensen's inequality*) in CAT. Therefore, the purpose of this brief report was to compare three methods of calculating marginalizing observed standard error (OSE) that can be expressed by the inverse of the test information function to estimate CAT reliabilities under varied test lengths. True reliability in classical test theory (CTT) is defined as the consistency or reproducibility of test score results, which is equivalent to the squared correlation between the true score (T) and the observed score (X), $\rho_{TX}^{2}$ and the squared correlation between observed scores from two parallel-forms (*X* and *X*′), $\rho_{XX^{\prime }}^{2}$(Crocker and Algina, 1986). Likewise, from the IRT perspective, θs are considered as true scores and $\hat{\theta }$s are considered as observed scores. Therefore, true reliability in IRT can be defined as the squared correlation between θs and $\hat{\theta }$, $\rho_{\left(\hat{\theta }\theta \right)}^{2}$. The mathematical form of the three-parameter logistic model (3PLM; Bock and Lieberman, 1970) is written as:

(1) $$\begin{array}{r} P_{ij}=c_{i}+\left(1-c_{i}\right)\frac{\exp\left[1.7a_{i}\left(\theta_{j}-b_{i}\right)\right]}{1+\exp\left[1.7a_{i}\left(\theta_{j}-b_{i}\right)\right]}, \end{array}$$

where *P*_*ij*_ is the probability of correctly answering item *i* given θ for examinee *j*, θ_*j*_ is the latent ability for examinee *j, b*_*i*_ is the item difficulty parameter for item *i, a*_*i*_ is the item discrimination parameter for item *i, c*_*i*_ is the pseudo-guessing parameter for item *i*. True reliability, however, cannot be computed in practical settings because true θs are unknown. Nevertheless, an empirical IRT reliability estimates, the square of the correlation between observed and true score ($\rho_{\left(\hat{\theta }\theta \right)}^{2}$), can be derived from the definition of CTT reliability (Lord and Novick, 1968; Green et al., 1984) as

(2) $${\hat{\rho }}_{\theta \hat{\theta }}^{2}=\frac{\left(\sigma_{\hat{\theta }}^{2}-(\sigma_{e|\hat{\theta }}^{2})\right)}{\sigma_{\hat{\theta }}^{2}},$$

where $\sigma_{\hat{\theta }}^{2}$ is the variance of $\hat{\theta }$ for all examinees and ${\bar{\sigma }}_{e|{\hat{\theta }}_{j}}^{2}$ is the mean of squared OSE for $\hat{\theta }$.

OSE can be computed by taking inverse of squared root of second derivative of likelihood function whenθ is estimated by MLE or MAP. The OSE is described as

(3) $$\begin{array}{r} \sigma_{e|{\hat{\theta }}_{j}}^{2}=\frac{1}{\sqrt{-\left(\frac{\partial^{2}\ln\;L\left(\text{u}|\theta_{j}\right)}{\partial \theta_{j}^{2}}\right)}}, \end{array}$$

where,

(4) $$\begin{array}{r} \left(\frac{\partial^{2}\ln\;L\left(\text{u}|\theta_{j}\right)}{\partial \theta_{j}^{2}}\right)=-\overset{n}{\underset{i\;=\;1}{\sum }}a_{i}^{2}P_{ij}Q_{ij} \end{array}$$

Equation (4) is equal to the test information function $I\left({\hat{\theta }}_{j}\right)$. Therefore, variance of OSE can be expressed by the test information function, $I\left({\hat{\theta }}_{j}\right)$, as follows:

(5) $$\begin{array}{r} \sigma_{e|{\hat{\theta }}_{j}}^{2}=\frac{1}{I\left({\hat{\theta }}_{j}\right)}, \end{array}$$

Based on Equation (5), this report applied three methods of marginalizing the variance of OSE (${\hat{\sigma }}_{e|\theta_{j}}^{2}$) for each examinee to estimate CAT reliability.

(1) *arithmetic mean:* $E_{\theta }\left(\sigma_{e|\hat{\theta }}^{2}\right)$was used to approximate CAT reliability as below:

(6) $${\hat{\rho }}_{1}^{2}=\frac{\left(\sigma_{\hat{\theta }}^{2}-E_{\theta }(\sigma_{e|\hat{\theta }}^{2})\right)}{\sigma_{\hat{\theta }}^{2}},$$

Note that, if $\hat{\theta }$ is the maximum likelihood estimate for each θ, then $\hat{\theta }$ will have a normal distribution with mean θ and asymptotical variance, 1/*I*(θ), where *I*(θ) is the test information function for each examinee based on IRT model (Samejima, 1994). In CAT, each examinee's θ has been estimated by different item pools so that $\sigma_{e|{\hat{\theta }}_{j}}^{2}$ is described for each examinee as below

(7) $$\begin{array}{r} \sigma_{e|{\hat{\theta }}_{j}}^{2}=E\left({\left({\hat{\theta }}_{j}-\theta_{j}\right)}^{2}|\theta_{j}\right)\approx \frac{1}{I\left(\theta_{j}\right)}, \end{array}$$

and remind that we assume *E*(*e*) = 0, and then mean of $\sigma_{e|\hat{\theta }}^{2}$ can be expressed by the mean of 1/$I\left(\hat{\theta }\right)$ as follows:

(8) $$\begin{array}{rcl} E_{\theta }\left(\sigma_{e|\hat{\theta }}^{2}\right) & = & E\left[e^{2}-E^{2}\left(e\right)\right]=E\left(e^{2}\right)=E_{\theta }\left[E\left(e^{2}|\theta \right)\right]\\ =E_{\theta }\left[E{\left(\hat{\theta }-\theta \right)}^{2}|\theta \right]\approx E_{\theta }\left[\frac{1}{I\left(\theta \right)}\right]. \end{array}$$

As a result, the mean of $\sigma_{e|\hat{\theta }}^{2}$ is actually approximated (Samejima, 1994). As

(9) $$\begin{array}{r} E_{\theta }\left(\sigma_{e|\hat{\theta }}^{2}\right)=\frac{\int \frac{1}{I\left(\theta \right)}g\left(\theta \right)d\theta }{\int g\left(\theta \right)d\theta }, \end{array}$$

where *g*(θ) is a density for the distribution of θ. In Equation (9), $\sigma_{e|\hat{\theta }}^{2}$ can be approximated by 1/*I*(θ).

(2) *harmonic mean*: ${\left(E\left(\sigma_{e|\hat{\theta }}\right)\right)}^{2}$ was used to approximate the mean variance of OSE, the second type of reliability can be approximated as below:

(10) $${\hat{\rho }}_{2}^{2}=\frac{\left[\sigma_{\hat{\theta }}^{2}-E_{\theta }{\left(\sigma_{e|\hat{\theta }}\right)}^{2}\right]}{\sigma_{\hat{\theta }}^{2}},$$

In similar to the first type of approximation, the second type of approximation is also described the test information as below:

(11) $$\begin{array}{r} E_{\theta }{\left(\sigma_{e|\hat{\theta }}\right)}^{2}={\left[\frac{\int \frac{1}{\sqrt{I\left(\theta \right)}}g\left(\theta \right)d\theta }{\int g\left(\theta \right)d\theta }\right]}^{2}, \end{array}$$

and

(3) *Jensen's Inequality* (see Rao, 1965):

${\left(\sigma_{e|\hat{\theta }=0}\right)}^{2}$, where $\sigma_{e|\hat{\theta }=0}$ is the OSE with $\hat{\theta }=0$, was used to marginalize ${\hat{\sigma }}_{e|\hat{\theta }}^{2}$. As a result, the third type of reliability can be approximated as below:

(12) $${\hat{\rho }}_{3}^{2}=\frac{\left[\sigma_{\hat{\theta }}^{2}-{\left(\sigma_{e|\hat{\theta }\;=\;0}\right)}^{2}\right]}{\sigma_{\hat{\theta }}^{2}}.$$

## Methods

### Test program

The item pool was created from the Emergency Medical Technician (EMT) exams administrated from 1/1/2013 to 9/1/2014. Based on the EMT practice analysis, 17~21% items of the test were assigned to Airway, Respiration, and Ventilation (ARV),16~20% items were assigned to Cardiology & Resuscitation (CR), 19~23% items were assigned to Trauma (TRA), 27~31% were assigned to Obstetrics and Gynecology (MOG) content, and 12%~16% were assigned to EMS operations (OPS) contents. The EMT operational item pool was composed of items that were previously calibrated using data from the paper-and-pencil tests and new items that were filed as tested in a previous CAT. The item pool has 1,136 items. The mean of item difficulty parameters for the item pool was 0.969. The item selection algorithm and content-balanced procedure proposed by Kingsbury and Zara (1989) was applied to this study. The CAT algorithm randomly selects the content area during the first 5 items and then content area that is most divergent from targeted percentage is selected next to meet the test plan (Kingsbury and Zara, 1989).

### Data simulation

**Figure 1:**
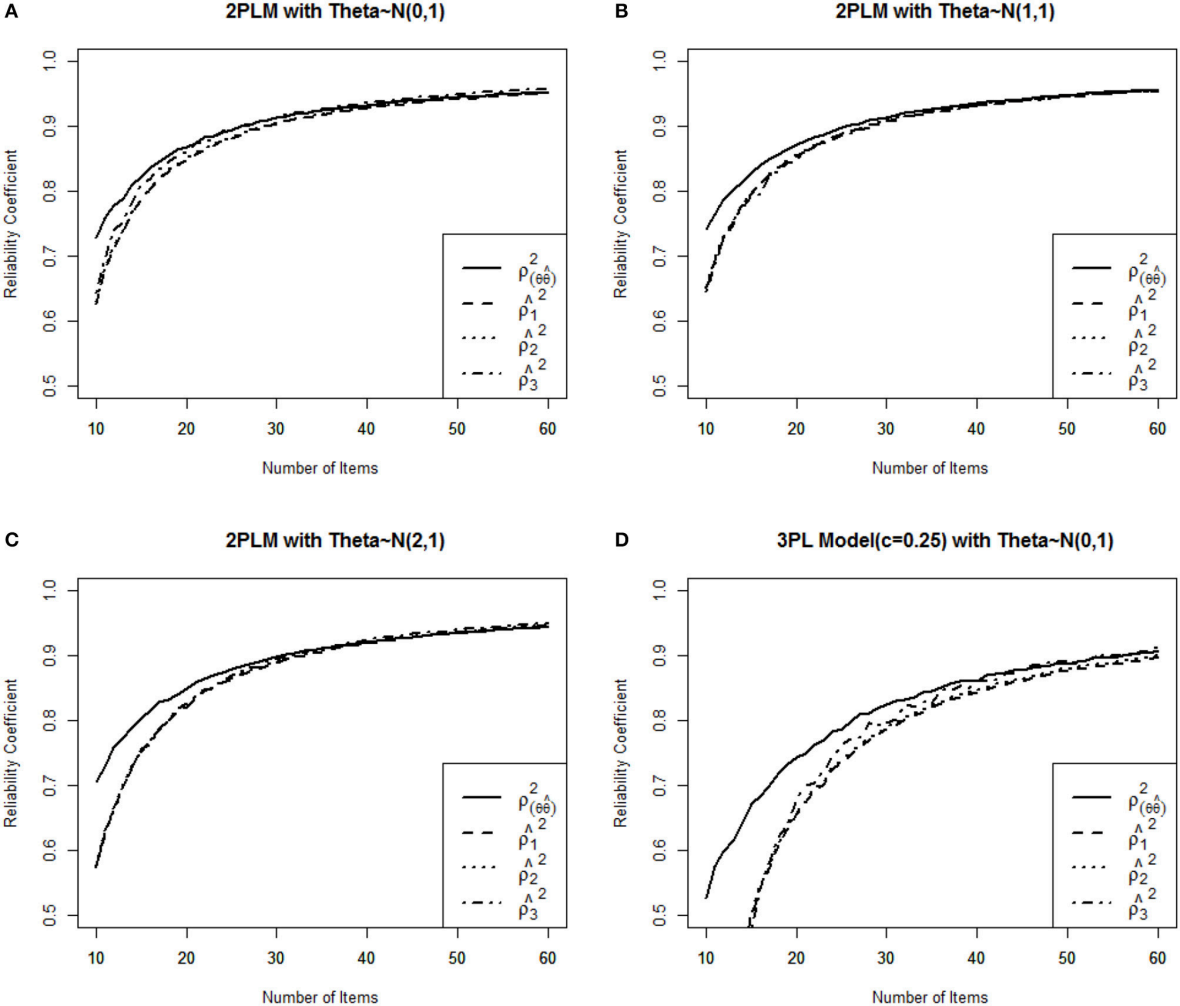
Comparison of three IRT reliability estimates with the true reliability for four different item pools. **(A)** 2PLM Medium Ability Group, **(B)** 2PLM High Ability Group, **(C)** 2PLM Extreme High Ability Group, **(D)** 3PLM Medium Ability Group.

The dichotomous IRT model (Bock and Lieberman, 1970) was applied to generate item responses with three examinee populations [N(0,1), N(1,1), and N(2,1)]. The *a*-parameters were generated from the mean of 1.0 and SD of 0.2 with *D* = 1.7, and *b*-parameter was from the item pool in 2PLM conditions, and *c*-parameter was set to 0.25 to evaluate the 3PLM conditions. To generate responses for each test, IRT model-based probabilities were compared to random numbers from a uniform distribution to obtain the item responses for each examinee. If the model-based probability was greater than the random number, the response to that item was recorded as correct (1). Otherwise, the item response was recorded as incorrect (0). This process was repeated for each item and examinee to obtain the full item response matrix for each item pool. A total of 1,000 examinees for each pool were generated with true θs following N(0,1), N(1,1), and N(2,1) using *D* = 1.7. In Figure 1A condition describes 2PL model with θs following N(0,1), (Figure 1B) condition describes 2PL model with θs following N(1,1), (Figure 1C) condition represents 2PL model with θs following N(2,1), and (Figure 1D) condition is designed for 3PL model with θs following N(0,1). For CAT termination, the fixed test length termination criteria were varied from 10 to 60 items within 1,136 item pool. To estimate stable CAT reliability estimates, each pool was replicated 100 times and average empirical reliabilities were calculated for each condition. Then average reliability was plotted as the fixed test length termination criteria were increased from 10 to 60 items. $\hat{\theta }$s and OSE of 1,000 examinees were estimated using MLE method. The “true” IRT reliabilities were computed as the squared correlation between the θ sand $\hat{\theta }$s($\rho_{\left(\hat{\theta }\theta \right)}^{2}$). The three empirical CAT reliabilities were obtained using *arithmetic mean, harmonic mean*, and *Jensen's inequality* respectively. Ability estimates were calculated using a Bayesian procedure until at least one item was answered correctly and one item was answered incorrectly. At that point, the ability estimates were calculated using MLE method. The Newton-Raphson procedure identified the maximum of the likelihood using an iterative procedure to estimate θ for MLE method. The Newton-Raphson iterations continued until the incremental change in $\hat{\theta }$ became less than the criterion of 0.001. Maximum Fisher information(MFI) was used as an item selection method in this study. MFI selects the next item that provides the maximum Fisher information at $\hat{\theta }$. All CAT algorithms for this study were implemented by a “catR” package (Magis and Raiche, 2012) in the R program (R Development Core Team, 2008).

## Results

Figure 1 shows the function of three empirical CAT reliabilities given four different conditions. As expected, CAT reliabilities became greater as the number of items increased as termination criterion, and then this study empirically shows that ${\hat{\rho }}_{1}^{2}\leq {\hat{\rho }}_{2}^{2}\leq {\hat{\rho }}_{3}^{2}$, as $E\left[\frac{1}{I\left(\theta \right)}\right]\geq {\left[E\left(\frac{1}{\sqrt{I\left(\theta \right)}}\right)\right]}^{2}\geq {\left(\frac{1}{\sqrt{I\left(\theta =0\right)}}\right)}^{2}$ in the Figures 1A,D (If we assume *I*(θ) is concave and mean of θ is 0). Overall, ${\hat{\rho }}_{1}^{2}$, ${\hat{\rho }}_{2}^{2}$, and ${\hat{\rho }}_{3}^{2}$ always underestimated true reliability except that ${\hat{\rho }}_{3}^{2}$ provided larger estimates after more than 30 items were administered for 2PLM and 50 items were administered for 3PLM (Figures 1A,D), and three reliability estimates were not differed to true reliability by more than .01 when the number of items administered was over 30 items for 2PLM. In terms of population ability, three estimates were almost identical to each other and were closer to true reliability when the mean of item difficulty parameters was equal to the mean of group abilities (Figure 1B) compared to two other population groups. ${\hat{\rho }}_{1}^{2}$, ${\hat{\rho }}_{2}^{2}$ and ${\hat{\rho }}_{3}^{2}$ were close to each other and consistent across all conditions, but ${\hat{\rho }}_{3}^{2}$ showed larger estimates rather than ${\hat{\rho }}_{1}^{2}$ and ${\hat{\rho }}_{2}^{2}$ when mean of θ is 0.0 (Figures 1A,B). Three reliability estimates were consistent across three conditions (Figures 1A–C), under the assumption that the 2PLM is true, which demonstrates the consistent results across different population abilities as an merit of CAT. In 3PLM, however, ${\hat{\rho }}_{1}^{2}$, ${\hat{\rho }}_{2}^{2}$, and ${\hat{\rho }}_{3}^{2}$ underestimated true reliability with the small number of items administered, and after more 50 items were administered, these estimates were not differed by more than 0.01 from the true reliability. Specifically, ${\hat{\rho }}_{3}^{2}$ showed larger estimates when only the mean of population was zero (Figures 1A,D), three reliability estimates were identical each other when the mean of population was equal to the mean of item difficulty in the item pool (Figure 1B). These results were not known in a previous research. Nicewander and Thomasson (1999) investigated CAT reliability with only 80 administered items with θ ranging −3 to +3 in 3PLM. However, longer than 50 items is not that in interesting in CAT setting. Table 1 showed that ${\hat{\rho }}_{3}^{2}$ overestimated the true reliability only if more than 50 items were administered in which mean of population ability was zero. This conclusion would hold when data are generated from 3PLM with the population mean of zero as known by Nicewander and Thomasson's study.

**Table 1:** Mean of three CAT reliability estimates with the true reliability for four different item pools.

**No of items admin**.	**2PLM with θ~ N(0,1)**				**2PLM with θ~N(1,1)**				**2PLM with θ~N(2,1)**				**3PL with θ~N(0,1), *c* = 0.25**			
	**$\rho_{\left(\hat{\theta }\theta \right)}^{2}$**	**${\hat{\rho }}_{1}^{2}$**	**${\hat{\rho }}_{2}^{2}$**	**${\hat{\rho }}_{3}^{2}$**	**$\rho_{\left(\hat{\theta }\theta \right)}^{2}$**	**${\hat{\rho }}_{1}^{2}$**	**${\hat{\rho }}_{2}^{2}$**	**${\hat{\rho }}_{3}^{2}$**	**$\rho_{\left(\hat{\theta }\theta \right)}^{2}$**	**${\hat{\rho }}_{1}^{2}$**	**${\hat{\rho }}_{2}^{2}$**	**${\hat{\rho }}_{3}^{2}$**	**$\rho_{\left(\hat{\theta }\theta \right)}^{2}$**	**${\hat{\rho }}_{1}^{2}$**	**${\hat{\rho }}_{2}^{2}$**	**${\hat{\rho }}_{3}^{2}$**
10 to 20	0.814	0.768	0.771	0.785	0.820	0.780	0.781	0.774	0.794	0.730	0.732	0.731	0.654	0.453	0.456	0.470
21 to 30	0.897	0.884	0.886	0.895	0.899	0.889	0.890	0.887	0.880	0.866	0.867	0.870	0.791	0.737	0.740	0.756
31 to 40	0.925	0.919	0.920	0.928	0.928	0.924	0.924	0.923	0.913	0.908	0.909	0.912	0.848	0.822	0.825	0.836
41 to 50	0.940	0.937	0.938	0.944	0.943	0.941	0.941	0.940	0.930	0.929	0.930	0.934	0.880	0.864	0.867	0.880
51 to 60	0.950	0.948	0.949	0.954	0.953	0.952	0.952	0.951	0.941	0.942	0.943	0.946	0.900	0.888	0.892	0.901

## Discussion

This brief report demonstrated that if the number of items administered was over 30, ${\hat{\rho }}_{1}^{2}$,${\hat{\rho }}_{2}^{2}$, and ${\hat{\rho }}_{3}^{2}$ provided accurate CAT reliability estimates for 2PLM. However, if the number of items administered in 3PLM was less than around 40 in this study, all three ${\hat{\rho }}_{1}^{2}$, ${\hat{\rho }}_{2}^{2}$, and ${\hat{\rho }}_{3}^{2}$ were relatively low. All three ${\hat{\rho }}_{1}^{2}$, ${\hat{\rho }}_{2}^{2}$, and ${\hat{\rho }}_{3}^{2}$ would be appropriate to report CAT reliability using all IRT models when over 50 items were administered in this study. However, including *c-*parameter brings higher OSE of $\hat{\theta }$ so that does not guarantee accurate reliability estimates when the number of items administered was less than 40 (differed by more than 0.02 from the true reliability). Although the 3PLM fits the data well, it does not accurately estimate person ability because *c*-parameter could inflate random error variance for examinee scoring (Chiu and Camilli, 2013). As a result, it was not recommended for reporting CAT reliability using 3PLM when a small number of items were administered. Compared with Nicewander and Thomasson (1999)'s study, this study demonstrated that three reliability estimates are appropriate to report CAT reliability regardless of ability population distributions and any IRT models if the number of items were administered from around 40 to 50 in CAT. They were differed within .01 from true reliability.

In summary, although reporting all three reliability estimates would be suggested regardless of any ability population distribution, ${\hat{\rho }}_{3}^{2}$is recommended for computing CAT reliability when mean of ability population distribution is 0 because ${\hat{\rho }}_{3}^{2}$ was closer to true reliability even if small number of items was administered and it can be computed easily by using a single test information value at θ = 0 in this study. In usual, a CAT was known as efficient and compared to a fixed-form test. However, a small fixed number of items was not suggested as a CAT termination criterion for 2PLM, specifically for 3PLM, in order to maintain high reliability estimates.

As with any research, this study has some limitations. This study examined the accuracy of CAT reliabilities under specific conditions for a medical licensing examination. Thus, there is a limitation to generalize this result to other testing conditions. Future studies would be needed to investigate the accuracy of CAT reliabilities under various conditions such as different ability distributions and item banks with different item parameter conditions.

## Author contribution

DS is the first author who conceptualize and write this brief research report and SJ is the corresponding author who manages this research project.

### Conflict of interest statement

The authors declare that the research was conducted in the absence of any commercial or financial relationships that could be construed as a potential conflict of interest.
